# Surfactant catalyzed convenient and greener synthesis of tetrahydrobenzo[*a*]xanthene-11-ones at ambient temperature

**DOI:** 10.3762/bjoc.7.9

**Published:** 2011-01-13

**Authors:** Pravin V Shinde, Amol H Kategaonkar, Bapurao B Shingate, Murlidhar S Shingare

**Affiliations:** 1 Department of Chemistry, Dr. Babasaheb Ambedkar Marathwada University, Aurangabad-431 004, India

**Keywords:** green chemistry, in-water synthesis, surfactant, tetradecyltrimethylammonium bromide (TTAB), xanthene

## Abstract

An efficient and greener protocol for the synthesis of 12-aryl-8,9,10,12-tetrahydrobenzo[*a*]xanthen-11-one using tetradecyltrimethylammonium bromide (TTAB) at room temperature in water is described.

## Introduction

The development of novel synthetic methodologies to facilitate the preparation of specific molecules is an intense area of research. In this regard, efforts have been constantly made to introduce new methodologies that are efficient and more compatible with the environment. One of the most desirable approaches to address this challenge is a search for surrogates for commonly employed organic solvents from various health and environmental reasons [1]. From the green chemistry point of view, water would be the perfect solvent to carry out chemical operations since it is safe, non-toxic, inexpensive and poses no threat to the environment [2]. However, water is rarely used or even considered as a solvent for organic reactions. One of the principal reasons is undoubtedly the limited solubility of most organic compounds in pure water. Since solubility is important for good reactivity, alternatives for improving the solubility of organic substrates that may ultimately help in expanding the scope of water-based organic syntheses have been investigated [3]. Incorporation of surface-active agents (surfactants) in aqueous media has been proved to enhance the reactivity of water mediated reactions via the formation of micelles or vesicular cavities. The use of micellar and vesicle forming surfactants as catalysts in water is widespread and has been studied for a number of different synthetic transformations/ multicomponent reactions in water [4].

Multicomponent reactions (MCRs) have emerged as an extremely powerful tool in combinatorial chemistry and drug discovery, since they offer significant advantages over conventional linear stepwise syntheses, in terms of improving classical organic reactions, for promoting new reactions and for the development of straightforward synthetic routes to bioactive heterocycles [5].

Xanthenes and benzoxanthenes constitute important classes of biodynamic heterocycles and their synthesis has received much attention especially in the field of medicinal/pharmaceutical chemistry due to their wide range of biological/pharmacological activities, e.g., antibacterial [6], anti-inflammatory [7] and antiviral [8]. Some xanthene based compounds have found application as antagonists for inhibiting the action of zoxalamine and in photodynamic therapy [9–10]. In addition, their derivatives can be used as dyes [11–12], pH sensitive fluorescent materials for the visualization of biomolecular assemblies [13] and in laser technologies [14–15].

Among the xanthene based compounds, tetrahydrobenzo[*a*]xanthene-11-ones are of interest and have great potential for further synthetic transformations [16–17]. Some novel methods for the synthesis of tetrahydrobenzo[*a*]xanthene-11-ones via multicomponent condensation reaction have been developed and catalysts such as NaHSO_4_.SiO_2_ [18], strontium triflate [19], Zr(HSO_4_)_4_ [20], dodecatungstophosphoric acid (PWA) [21], iodine [22], InCl_3_/P_2_O_5_ [23] and *p*-toluenesulfonic acid/ionic liquid([bmim]BF_4_) [24] have been employed for their synthesis. However, in an era where green methods are desirable many of these methods are unsatisfactory as they involve the use of halogenated solvents, catalyst loadings of up to 30 mol %, low yields, drastic reaction conditions, prolonged reaction times and tedious isolation procedures. All of these disadvantages make further improvements for the synthesis of such molecules essential. Recently, synthetic methods that involve tetra(*n*-butyl)ammonium fluoride (TBAF) [25] and proline triflate [26] in water have been described. However, the major problems associated with these routes are the need for higher/reflux conditions and longer reaction times. Therefore, it was thought worthwhile to develop a new greener and more convenient method for the preparation of tetrahydrobenzo[*a*]xanthene-11-ones.

Considering the significance of surfactants and in continuation of our program [27–33] to develop new and convenient synthetic protocols for the construction of bioactive heterocycles, herein we wish to report a highly efficient synthesis of 12-aryl-8,9,10,12-tetrahydrobenzo[a]xanthen-11-ones using tetradecyltrimethylammonium bromide (TTAB) in aqueous micellar form. The pronounced catalytic effect of TTAB in organic synthesis is described for the first time.

## Results and Discussion

**Scheme 1:**
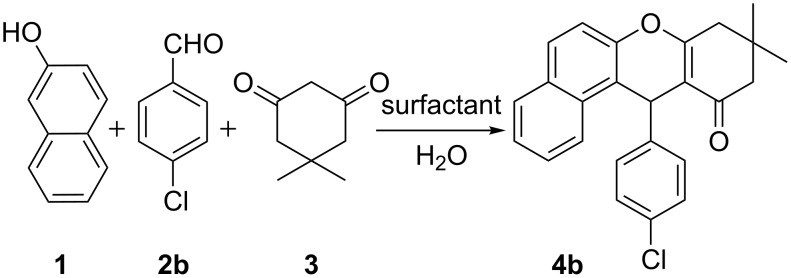
Standard model reaction.

In our initial study, reaction of 4-chlorobenzaldehyde, β-naphthol and dimedone in water was considered as a standard model reaction (Scheme 1). During this investigation, efforts were mainly focused on a variety of surfactants. In this regard, different cationic surfactants such as cetyltrimethylammonium bromide (CTAB), methyltriphenylphosphonium bromide (MTPPB) and cetylpyridinium chloride (CPC) as well as an anionic surfactant, sodium dodecyl sulfate (SDS), were utilized at ambient temperature.

From these preliminary studies, it was observed that the anionic surfactant SDS and cationic surfactants CPC and MTPPB gave the desired product albeit in low yields, i.e., 57%, 32% and 59% respectively (Table 1, entries 1–3). In contrast, the cationic surfactant CTAB accelerated the model reaction to afford the desired product in good 81% yield (Table 1, entry 4). From this, it was concluded that cationic surfactants, particularly quaternary ammonium where the counterion is bromide are far superior to other surfactants for efficient catalysis.

**Table 1:** Screening of surfactants.^a^

Entry	Surfactant	Temperature (°C)	Time (h)	Yield^b^ (%)

1	SDS	RT^c^	4	57
2	CPC	RT^c^	4	32
3	MTPPB	RT^c^	4	59
4	CTAB	RT^c^	3	81
5	TEAB	RT^c^	4	43
6	TBAB	RT^c^	4	68
7	TTAB	RT^c^ (2.5, 5, 10, 15, 20)^d^	2.5	55, 72, 85, 88, 89
8	TTAB	60	3	85
9	TTAB	80	3	81
10	TTAB	100	3	76
11	TTAB	Reflux	3	71

^a^reaction conditions: **1** (1 mmol), **2b** (1 mmol), **3** (1 mmol), surfactant (10 mol %), in water (5 mL); ^b^isolated yields; ^c^room temperature (RT) was 40 °C; ^d^numbers in parentheses indicate concentration of surfactant and corresponding yields are given in the "Yield" column.

Encouraged by these results, we then investigated some more cationic surfactants, particularly quaternary ammonium bromides. For this purpose, we utilized TEAB, TBAB and TTAB in the model reaction. After a careful study, decreased reaction times and increased product yields were observed with increasing alkyl chain length of the surfactant up to a C_14_ chain length. Longer alkyl chains led to a slight decrease in product yield. TEAB did not afford more than 43% yield of product, even after 4 h (Table 1, Entry 5), whereas TBAB gave a 68% yield (Table 1, Entry 6). By comparison, TTAB influenced both the yield and the reaction time and gave the product in an excellent 85% yield (Table 1, Entry 7) within only 2.5 h and proved to be a better catalyst than CTAB. The success of TTAB as an efficient catalysis could be related to the number of carbon atoms in the hydrophobic alkyl chain of surfactant, which reaches saturation, in this case, C_14_, after which the reaction yield and reaction time are independent of the surfactant with alkyl chains larger than C_14_.

**Figure 1:**
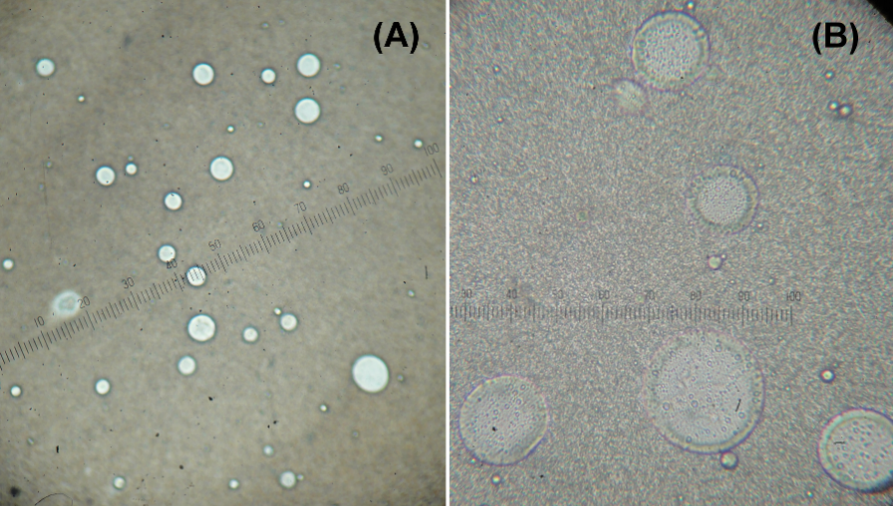
Optical micrograph of the reaction mixture. (A) normal view, (B) magnified view. (Scale bar = 2.5 µm).

It is of note to point out that the addition of TTAB converted the initially floating reaction mass into a homogeneous mixture, which on stirring became a white turbid emulsion. This observation implies, that there was formation of micelles or micelle-like colloidal aggregates. Indeed, formation of spherical droplets in water was confirmed by optical microscopy [34–35] (Figure 1).

**Figure 2:**
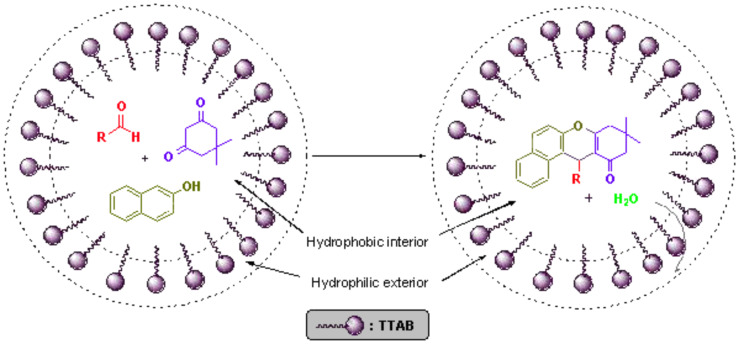
Schematic diagram representing the role of TTAB.

It is well known that dehydration reactions are difficult to carry out in water, since water generated during the reaction needs to be removed to shift the equilibrium towards the side of the dehydrated product. Nevertheless, by introducing a surfactant (TTAB), dehydration was successfully achieved in water. The catalytic effect of the micellar solution of TTAB may be attributed to the hydrophobic nature of organic substrates. Formation of emulsion droplets takes place in water in the presence of surfactant and substrate molecules. It is suggested that most of the organic substrates are concentrated in these spherical droplets, which act as a hydrophobic reaction sites and results in an increase in the effective concentration of the organic reactants, which might increase the reaction rate via a concentration effect. In micellar solution, organic substrates are pushed away from water molecules towards the hydrophobic core of micelle droplets thus inducing efficient collisions between organic substrates which eventually enhance the reaction rate and result in rapid reactions in water. The hydrophobic interior of the micelles swiftly excludes the water molecules generated during the reaction, thus shifting the equilibrium towards the desired product that ultimately leads to an increase in the reaction yield [34–36]. This explanation is schematically represented in Figure 2.

We next investigated the effect of temperature on the rate of reaction. For this purpose the reaction was carried at higher temperatures, i.e., 60 °C, 80 °C, 100 °C and under reflux conditions. However, increasing the temperature failed to enhance the reaction rate substantially. In point of fact, higher temperatures lowered the product yield slightly, accompanied by some impurities (Table 1, Entries 8–11).

Catalyst concentration is a significant factor that exclusively affects the reaction rate and product yield. To study this, the reaction was performed at different concentrations of TTAB, i.e., 2.5, 5, 10, 15, and 20 mol %, and gave the product in 55%, 72%, 85%, 88% and 89% yield, respectively (Table 1, entry 7). Thus, it was clear that reaction rate increased with increasing catalyst concentration up to 15 mol % without any significant difference on further increasing the catalyst concentration. It means 15 mol % of surfactant was sufficient for catalyzing the reaction effectively.

In accordance with the literature [15], a plausible mechanistic path for the formation of tetrahydrobenzo[*a*]xanthen-11-ones can be outlined as follows: nucleophilic addition of 2-naphthol to the aldehyde to give an intermediate *ortho*-quinone methide (*o*-QM), subsequent Michael addition of dimedone to the *o*-QM followed by attack of the phenolic –OH group of the *o*-QM at the carbonyl carbon of dimedone to yield a cyclic hemiketal that on dehydration affords the final product.

To generalize the synthetic procedure, various electronically divergent aryl aldehydes were treated with β-naphthol and dimedone under the optimized reaction conditions and all these substrates were found to be equally amenable to these conditions. Interestingly, some heteroaryl aldehydes also underwent the reaction smoothly. Representative results are summarized in Table 2. Formation of the products was confirmed by IR, ^1^H NMR, ^13^C NMR and mass spectrometry.

**Table 2:** Synthesis of 12-aryl-8,9,10,12-tetrahydrobenzo[*a*]xanthen-11-ones.^a^

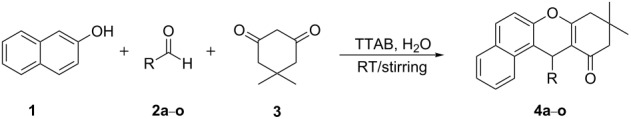					

Entry	Comp.	R	Time (h)	Yield^b^ (%)	mp [ref.] (°C)

1	**4a**	Ph	3	87	150–151 [20]
2	**4b**	4-Cl-C_6_H_4_	2.5	88	182–184 [20]
3	**4c**	4-Me-C_6_H_4_	3	85	174–176 [20]
4	**4d**	4-MeO-C_6_H_4_	3.5	89	205–206 [20]
5	**4e**	4-F-C_6_H_4_	2.5	85	184–186 [22]
6	**4f**	2-Cl-C_6_H_4_	3	84	177–178 [20]
7	**4g**	2-NO_2_-C_6_H_4_	2	89	222–224 [20]
8	**4h**	3-NO_2_-C_6_H_4_	3	89	169–170 [20]
9	**4i**	4-NO_2_-C_6_H_4_	2.5	91	180–181 [20]
10	**4j**	4-HO-C_6_H_4_	3	88	221–223 [20]
11	**4k**	4-Br-C_6_H_4_	3	87	187–189 [19]
12	**4l**	Piperonyl	4	91	211–212 [22]
13	**4m**^c^	2-Thienyl	6	79	176–178^c^
14	**4n**^c^	2-Furfuryl	6	74	170–172^c^
15	**4o**^c^	3-Indolyl	8	66	202–204^c^

^a^reaction conditions: **1** (1 mmol), **2** (1 mmol), **3** (1 mmol), TTAB (15 mol %) in water (5 mL) at RT (40 °C); ^b^isolated yields; **^c^**formation of these compounds were confirmed on the basis of spectral analyses.

## Conclusion

In conclusion, we have developed an exceedingly simple, mild and clean synthetic protocol for the synthesis of tetrahydrobenzo[*a*]xanthene-11-ones. In this method, the use of TTAB for an organic transformation has been described for the first time. Water is not only an inexpensive and environmentally benign solvent, but also plays an important role in reactivity and selectivity. Surfactants catalyze the reaction efficiently at room temperature with short reaction times without using any harmful organic reagents and solvents.

## Experimental

**General:** All chemicals were purchased and used without any further purification. Melting points were determined on a Veego apparatus and are uncorrected. Infrared spectra were recorded on a Bruker spectrophotometer as KBr discs, and the absorption bands are expressed in cm^−1^. ^1^H NMR and ^13^C NMR spectra were recorded on an NMR spectrometer AC200 in CDCl_3_, chemical shifts (δ) are given in ppm relative to TMS. Mass spectra were taken on a Macro mass spectrometer (Waters) by the electrospray method (ES).

**Optical microscopy measurements:** A drop of the turbid reaction mixture was diluted with distilled water, and then subjected to light microscopy measurement using an ordinary light microscope under 400× magnification.

**Typical experimental procedure:** To a mixture of β-naphthol **1** (0.144 g, 1 mmol), 4-chlorobenzaldehyde **2b** (0.140. g, 1 mmol) and dimedone **3** (0.140 g, 1 mmol) in water (5 mL), was added TTAB (0.050 g, 15 mol %). This reaction mixture was allowed to stir vigorously at room temperature. Progress of the reaction was monitored by TLC (ethyl acetate:*n*-hexane = 2:8). After completion of the reaction (2.5 h), the solid obtained was collected by filtration and washed successively with warm water and aqueous ethanol. The crude product was recrystallized from ethanol to afford the pure product which required no further purification. (It is important to note here that some crude products were obtained as sticky solids and in such cases, before isolation of product, they were treated with aqueous ethanol.)

**12-(4-chlorophenyl)-9,9-dimethyl-8,9,10,12-tetrahydrobenzo[*a*]xanthen-11-one** (**4b**) ^1^H NMR (CDCl_3_, 200 MHz): δ 1.00 (s, 3H), 1.16 (s, 3H), 2.29 (d, 2H, J = 4 Hz), 2.59 (d, 2H, J = 4 Hz), 5.70 (s, 1H), 7.17–7.43 (m, 7H, Ar-H), 7.77–7.89 (m, 3H, Ar-H); ^13^C NMR (50 MHz, CDCl_3_): δ 27.1, 29.3, 32.5, 34.3, 41.9, 50.9, 113.1, 116.8, 117.3, 123.9, 125.0, 127.2, 128.7, 129.4, 130.2, 131.5, 131.9, 132.6, 143.3, 145.1, 150.8, 164.2, 196.9; IR (KBr, cm^−1^): *v* 2952, 1648, 1597, 1373, 1231, 1184, 823; ES-MS: 389.14 [M^+^], 391.13 [M^+^ + 2].

**12-(2-nitrophenyl)-9,9-dimethyl-8,9,10,12-tetrahydrobenzo[*a*]xanthen-11-one** (**4g**) ^1^H NMR (CDCl_3_, 200 MHz): δ 0.87 (s, 3H), 1.10 (s, 3H), 2.19 (d, 2H, J = 4Hz), 2.52 (d, 2H, J = 4 Hz), 6.56 (s, 1H), 7.02 (d, 1H, J = 8 Hz), 7.18–7.46 (m, 5H, Ar-H), 7.75–7.88 (m, 3H, Ar-H), 8.51 (d, 1 H, J = 8 Hz); ^13^C NMR (50 MHz, CDCl_3_): δ 27.2, 29.5, 32.1, 34.6, 41.1, 51.4, 112.9, 116.6, 117.8, 124.1, 124.7, 126.9, 127.4, 128.1, 128.7, 129.5, 129.9, 131.3, 131.7, 132.2, 134.0, 141.5, 149.2, 163.9, 196.1; IR (KBr, cm^−1^): v 2957, 1651, 1595, 1537, 1376, 1348, 1226, 1174, 818; ES-MS: 400.16 [M^+^].

**12-[4-(benzo[*d*][1,3]dioxol-5-yl)]-9,9-dimethyl-8,9,10,12-tetrahydrobenzo[*a*]xanthen-11-one** (**4l**) ^1^H NMR (CDCl_3_, 200 MHz): δ 1.01 (s, 3H), 1.13 (s, 3H), 2.28 (s, 2H), 2.56 (s, 2H), 5.62 (s, 1H), 5.80 (d, 1H, J = 1.2 Hz) 5.84 (d, 1H, J = 1.2 Hz), 6.59 (d, 1H, J = 8 Hz), 6.77 (td, 2H, J = 8, 2 Hz), 7.26–7.48 (m, 3H), 7.72–7.79 (m, 2H), 7.95 (d, 1H, J = 8 Hz); ^13^C NMR (50 MHz, CDCl_3_): δ 26.9, 29.6, 32.6, 34.7, 41.4, 51.7, 101.5, 111.2, 113.8, 115.1, 117.6, 122.3, 123.8, 124.8, 128.3, 128.8, 129.4, 130.1, 131.6, 132.5, 140.0, 145.5, 146.7, 149.1, 163.4, 197.6; IR (KBr, cm^−1^): v 2948, 1639, 1592, 1368, 1234, 1178, 1039, 829; ES-MS: 399.21 [M^+^].

**12-(thiophen-2-yl)-9,9-dimethyl-8,9,10,12-tetrahydrobenzo[*a*]xanthen-11-one** (**4m**) ^1^H NMR (CDCl_3_, 200 MHz): δ 1.05 (s, 3H), 1.14 (s, 3H), 2.34 (s, 2H), 2.56 (s, 2H), 6.02 (s, 1H), 6.72–6.75 (m, 2 H), 6.99 (dd, 1H, J = 4Hz), 7.40–7.51 (m, 2H), 7.75–7.82 (m, 2H), 8.01 (d, 1H, J = 8 Hz); ^13^C NMR (50 MHz, CDCl_3_): δ 27.5, 29.4, 32.9, 34.8, 41.8, 51.2, 113.4, 115.2, 117.7, 123.0, 126.5, 127.2, 127.9, 129.6, 130.3, 130.8, 132.1, 132.6, 135.4, 139.7, 144.6, 162.7, 197.6; IR (KBr, cm^−1^): v 2947, 1638, 1593, 1372, 1225, 1061, 724; ES-MS: 361.13 [M^+^].

**12-(furan-2-yl)-9,9-dimethyl-8,9,10,12-tetrahydrobenzo[*a*]xanthen-11-one** (**4n**) ^1^H NMR (CDCl_3_, 200 MHz): δ 1.06 (s, 3H), 1.16 (s, 3H), 2.38 (s, 2H), 2.62 (s, 2H), 6.02 (s, 1H), 6.76–6.81 (m, 2 H), 7.01 (dd, 1H, J = 4Hz), 7.46–7.58 (m, 2H), 7.80–7.85 (m, 2H), 8.03 (d, 1H, J = 8 Hz); ^13^C NMR (50 MHz, CDCl_3_): δ 27.6, 29.6, 33.0, 34.9, 41.6, 51.3, 112.2, 114.7, 123.5, 123.9, 126.6, 127.1, 128.2, 128.8, 129.5, 129.9, 130.3, 132.2, 133.4, 136.1, 152.7, 162.9, 198.2; IR (KBr, cm^−1^): v 2951, 1641, 1596, 1362, 1231, 1058, 735; ES-MS: 345.09 [M^+^].

**12-(indol-3-yl)-9,9-dimethyl-8,9,10,12-tetrahydrobenzo[*a*]xanthen-11-one** (**4o**) ^1^H NMR (CDCl_3_, 200 MHz): δ 0.91 (s, 3H), 1.12 (s, 3H), 2.24 (d, 2H, J = 6 Hz), 2.61 (s, 2H), 6.00 (s, 1H), 6.98 (td, 2H, J = 8, 2 Hz), 7.16 (d, 2H, J = 4 Hz ), 7.31–7.37 (m, 3H), 7.48 (d, 1H, J = 8 Hz ), 7.71 (d, 2H, J = 8 Hz), 8.09 (d, 1H, J = 8 Hz), 8.16 (s, 1H, -NH); ^13^C NMR (50 MHz, CDCl_3_): δ 26.5, 28.9, 31.8, 34.2, 41.2, 51.0, 111.8, 114.1, 116.9, 117.7, 119.7, 120.2, 123.9, 124.2, 125.1, 126.1, 128.5, 129.2, 129.6, 130.7, 131.9, 135.4, 141.2, 148.8, 164.2, 197.1; IR (KBr, cm^−1^): *v* 3363, 1648, 1597, 1365, 1229, 1175, 1142, 796; ES-MS: 394.19 [M^+^].
